# Association between prognostic nutritional index and major adverse cardiovascular events in patients with atrial fibrillation combined with heart failure with preserved ejection fraction

**DOI:** 10.3389/fnut.2026.1707560

**Published:** 2026-04-01

**Authors:** Chunyang Xu, Zhihua Wang, Liping Liu

**Affiliations:** Department of Cardiology, Yancheng First Hospital, Affiliated Hospital of Nanjing University Medical School (Yancheng No.1 People’s Hospital), Yancheng, China

**Keywords:** atrial fibrillation, heart failure with preserved ejection fraction, major adverse cardiovascular events, prognostic nutritional index, risk prediction

## Abstract

**Objective:**

To investigate the association between the prognostic nutritional index (PNI) and major adverse cardiovascular events (MACE) and all-cause mortality in patients with atrial fibrillation (AF) and heart failure with preserved ejection fraction (HFpEF).

**Methods:**

A total of 734 consecutive patients with AF and HFpEF were included in this retrospective cohort study, which was conducted at Yancheng First Hospital from July 2022 to July 2025. Cox proportional hazards regression models were applied to evaluate the relationship between PNI with MACE and all-cause mortality, with additional subgroup analyses performed across major clinical strata. The discriminative ability of PNI was examined using receiver operating characteristic (ROC) curve analysis, and restricted cubic spline (RCS) modeling was used to examine dose–response patterns.

**Results:**

During a median follow-up of 35 months, 131 MACE were recorded (17.8%). In multivariable Cox regression, PNI analyzed as a continuous variable was inversely associated with MACE risk, with an adjusted hazard ratio (HR) of 0.904 (95% CI: 0.869–0.940); standardized PNI showed a similar and consistent association (HR = 0.578). Similarly, higher PNI was significantly associated with a reduced risk of all-cause mortality (*P* < 0.001). When assessed categorically by tertiles, patients in the highest PNI group (T3) had significantly lower risks of both MACE (HR = 0.366) and all-cause mortality (HR = 0.174) compared with the lowest tertile (*P* < 0.05). Similar inverse trends were observed across median, optimal cutoff, and quartile-based groupings (*P* < 0.05). Most subgroup analyses supported the inverse relationship between PNI and MACE (*P* < 0.05). ROC curve analysis showed that PNI demonstrated limited discriminatory ability for MACE with an area under the curve (AUC) of 0.654 (95% CI: 0.603–0.705). RCS analysis indicated a significant linear inverse association between PNI and MACE (P-overall < 0.001), with no evidence of non-linearity (P-nonlinear = 0.330).

**Conclusion:**

In patients with AF and HFpEF, higher PNI levels were independently associated with a reduced risk of MACE and all-cause mortality, indicating that PNI may serve as a potential adjunctive marker for risk stratification.

## 1 Introduction

Heart failure (HF) is a major contributor to morbidity and mortality worldwide. In recent years, heart failure with preserved ejection fraction (HFpEF) has received increasing attention in clinical practice, and its prognostic implications have become the focus of extensive research (1). Atrial fibrillation (AF) is one of the most frequent comorbidities in HFpEF (2). Evidence from numerous studies indicates a bidirectional relationship between AF and HFpEF: AF impairs atrial contraction and leads to uncontrolled ventricular rates, thereby worsening diastolic filling pressures (3). Conversely, the elevated left atrial load and atrial fibrosis seen in HFpEF promote the persistence of AF. This vicious cycle substantially increases the likelihood of rehospitalization and mortality. Consequently, patients with concomitant HFpEF and AF represent a distinct and particularly high-risk clinical subgroup. In both clinical research and practice, major adverse cardiovascular events (MACE) are commonly used as composite endpoints (4). MACE typically include cardiovascular death, non-fatal myocardial infarction (MI), stroke, unplanned revascularization, and cardiovascular-related rehospitalization. Such composite measures provide a comprehensive assessment of long-term outcomes in patients with cardiovascular disease (CVD). Despite recent advances in the diagnostic criteria and management strategies for HFpEF, achieving accurate, efficient, and reproducible risk stratification in patients with concurrent AF and HFpEF remains a significant challenge in routine care (5).

In recent years, malnutrition and inflammation have been increasingly recognized as non-traditional prognostic factors in HF (6–8). HFpEF patients, who are often elderly, frequently exhibit malnutrition, frailty, and chronic inflammatory activation, all of which have been consistently associated with adverse clinical outcomes (9, 10). Recent studies have also highlighted the prognostic significance of nutritional status in patients with non-valvular AF (NVAF). In particular, the AFTER-2 study demonstrated that nutritional indices—including the prognostic nutritional index (PNI) and hemoglobin, albumin, lymphocyte, platelet (HALP) score—were significantly associated with mortality risk in this population (11, 12). These findings further emphasize the need for simple and integrated markers that reflect both nutritional and inflammatory status. The PNI, calculated from serum albumin and lymphocyte count, provides such a marker by incorporating both nutritional and immunological components (13). Due to its simplicity and accessibility, PNI has gained increasing attention across various CVD as a predictor of adverse outcomes, including in HF (14–16). Existing studies in both acute and chronic HF cohorts have also shown that lower PNI is independently associated with higher risks of all-cause mortality and rehospitalization (17, 18). Furthermore, a recent meta-analysis demonstrated that patients with lower PNI had a markedly increased risk of MACE compared with those with higher PNI, with an adjusted hazard ratio (HR) of approximately 2.3 (19). However, evidence regarding the role of PNI in patients with AF combined with HFpEF is scarce. A recent study reported that PNI was a significant predictor of AF recurrence following catheter ablation in patients with non-valvular AF and HFpEF (20). Yet, this work primarily addressed arrhythmia recurrence rather than MACE. As such, the potential association between PNI and MACE in this high-risk subgroup remains largely unexplored.

Taken together, the coexistence of AF and HFpEF defines a population at elevated risk of MACE. PNI, as a readily obtainable nutritional–immune composite index, has shown promising prognostic relevance in HF and broader CVD populations. However, systematic evidence regarding its prognostic role specifically in patients with both AF and HFpEF is currently lacking. To our knowledge, limited data are available regarding the prognostic role of PNI in patients with coexisting AF and HFpEF. Importantly, the present study is not intended to establish a novel mechanistic pathway, but rather to validate and refine risk stratification in a clinically distinct and high-risk subgroup characterized by the coexistence of AF and HFpEF. Although the prognostic value of PNI has been reported in broader HF and AF populations, systematic evidence specifically addressing composite cardiovascular endpoints in patients with concurrent AF and HFpEF remains limited. Therefore, our study should be interpreted as a focused risk re-stratification analysis in a pathophysiologically complex subgroup rather than a replication of prior findings in general cardiovascular cohorts.

Given the above background, we hypothesize that lower PNI is independently associated with a higher risk of MACE and all-cause mortality in patients with AF and HFpEF. Therefore, the present study aims to investigate the relationship between baseline PNI and the incidence of MACE and all-cause mortality during follow-up. Importantly, we do not propose that restricting the analysis to a combined phenotype alone constitutes conceptual advancement. Instead, the clinical relevance of this study stems from the distinct pathophysiological interplay between AF and HFpEF, including atrial remodeling, diastolic dysfunction, inflammatory activation, and neurohormonal dysregulation. These interacting mechanisms create a risk profile that is not merely additive but structurally different from isolated HF or AF populations. Therefore, the present analysis should be interpreted as a targeted risk re-stratification effort within a biologically and clinically distinct high-risk subgroup.

## 2 Materials and methods

### 2.1 Study population

This investigation was a single-center retrospective cohort study that enrolled a total of 734 consecutive patients from the Department of Cardiology at Yancheng First Hospital between July 2022 and July 2025. The present study adhered to the ethical principles set forth in the Declaration of Helsinki. The study protocol was reviewed and approved by the Institutional Ethics Committee of Yancheng First Hospital (2025-K-229). Before enrollment, all participants provided informed consent. Patient information was anonymized for the analysis, with strict measures in place to protect confidentiality.

To be eligible, patients had to meet these inclusion criteria: (1) A confirmed diagnosis of AF; (2) Fulfillment of the diagnostic criteria for HFpEF; (3) Age ≥ 35 years. The exclusion criteria were as follows: (1) valvular AF, such as rheumatic mitral stenosis or AF after mechanical prosthetic valve replacement; (2) severe renal impairment [estimated glomerular filtration rate (Egfr) < 15 mL/min/1.73 m^2^] or end-stage liver failure; (3) significant hematologic disorders or active malignancy; (4) presence of acute infection or autoimmune disease at baseline; (5) cachexia or severe malnutrition; (6) incomplete clinical or laboratory data, particularly missing serum albumin or lymphocyte counts required for PNI calculation; (7) loss to follow-up.

### 2.2 Definition and grouping of prognostic nutritional index

The PNI at baseline was calculated for each patient using the formula: PNI = serum albumin (g/L) + 0.005 × peripheral lymphocyte count (per mm^3^) (13). To minimize the impact of scale differences between variables, PNI values were also standardized, expressed as: Standardized PNI = (PNI - mean)/standard deviation (SD).

In subsequent analyses, both raw and standardized values of PNI were examined as continuous measures and as a categorical variable. Patients were stratified according to several grouping strategies. Based on the median value of 44.71, the cohort was divided into a low-PNI group (PNI ≤ 44.71, *n* = 367) and a high-PNI group (PNI > 44.71, *n* = 367). Using the optimal cutoff of 43.36 derived from the receiver operating characteristic (ROC) curve analysis based on the maximization of the Youden index, patients were further categorized into low PNI (PNI ≤ 43.36, *n* = 290) and high PNI (PNI > 43.36, *n* = 444). According to tertile thresholds (42.30 and 46.84), three groups were defined: T1 (PNI ≤ 42.30, *n* = 246), T2 (42.30 < PNI ≤ 46.84, *n* = 244), and T3 (PNI > 46.84, *n* = 244). Finally, using quartile cutoffs (41.14, 44.71, and 48.05), patients were classified into four subgroups: Q1 (PNI ≤ 41.14, *n* = 183), Q2 (41.14 < PNI ≤ 44.71, *n* = 184), Q3 (44.71 < PNI ≤ 48.05, *n* = 185), and Q4 (PNI > 48.05, *n* = 182).

### 2.3 Clinical characteristics and study variables

Comprehensive clinical, laboratory, and echocardiographic data were collected and analyzed as baseline characteristics and potential covariates in this study. Demographic and lifestyle factors included age, gender, and smoking status. HF severity was assessed using the New York Heart Association (NYHA) functional classification, with classes I–II indicating mild symptoms and classes III–IV representing moderate-to-severe limitation.

AF was diagnosed when patients exhibited an irregularly irregular RR interval, absence of discernible P waves, and chaotic atrial activity on a standard 12-lead electrocardiogram (ECG) or continuous cardiac monitoring, consistent with generally accepted diagnostic criteria (21). ECG recordings were independently interpreted by at least one certified cardiologist. In cases of uncertainty, tracings were reviewed by a second senior cardiologist to ensure diagnostic accuracy and reduce misclassification bias. HFpEF diagnosis strictly followed the 2021 ESC guideline recommendations, and all patients fulfilled the three core criteria: (1) the presence of typical HF manifestations, including dyspnea, fatigue, or exercise intolerance; (2) a left ventricular ejection fraction (LVEF) ≥ 50%; and (3) objective evidence of diastolic dysfunction and/or elevated ventricular filling pressures. The latter included structural remodeling (such as a left atrial volume index > 34 mL/m^2^, left ventricular mass index ≥ 95 g/m^2^ in women or ≥ 115 g/m^2^ in men, or relative wall thickness > 0.42), functional alterations (including an E/e’ ratio > 9, tricuspid regurgitation velocity > 2.8 m/s, or pulmonary artery systolic pressure > 35 mmHg), and elevated biomarkers [N-terminal pro-B-type natriuretic peptide (NT-proBNP) > 365 pg/mL or BNP > 105 pg/mL] (22). All echocardiographic examinations were performed using standardized imaging protocols by experienced sonographers at our institution. LVEF was measured using the modified biplane Simpson’s method in accordance with guideline recommendations. In cases of suboptimal image quality or measurement discrepancies, results were reviewed and confirmed by a senior cardiologist with expertise in cardiac imaging to ensure measurement consistency. Because the study was conducted at a single center, imaging equipment, acquisition procedures, and interpretation standards were uniform, thereby minimizing inter-observer and inter-institutional variability.

Comorbidities were carefully defined as follows: hypertension was identified by a prior medical diagnosis, current use of antihypertensive therapy, or repeated office blood pressure measurements ≥ 140/90 mmHg (23); diabetes was defined as a documented history of diabetes, fasting blood glucose (FBG) ≥ 7.0 mmol/L, glycated hemoglobin (HbA1c) ≥ 6.5%, or ongoing antidiabetic treatment (24); dyslipidemia was defined as total cholesterol ≥ 6.2 mmol/L, triglycerides ≥ 2.3 mmol/L, low-density lipoprotein cholesterol (LDL-C) ≥ 4.1 mmol/L, high-density lipoprotein cholesterol (HDL-C) < 1.0 mmol/L, or treatment with lipid-lowering medication (25); coronary heart disease (CHD) was defined by a history of MI, prior percutaneous coronary intervention, or coronary artery bypass grafting; stroke included clinically documented ischemic or hemorrhagic events confirmed by imaging; and chronic kidney disease (CKD) was defined as an eGFR < 60 mL/min/1.73 m^2^ for at least 3 months or a confirmed prior diagnosis of CKD (26). Thromboembolic risk was assessed using the CHA_2_DS_2_-VASc score.

Anthropometric measurements included body mass index (BMI), while vital signs comprised resting heart rate, systolic blood pressure (SBP), and diastolic blood pressure (DBP) at admission. Hematological parameters covered white blood cell (WBC), neutrophil, lymphocyte, and monocyte counts, along with hemoglobin and platelet levels. Biochemical tests included serum albumin, uric acid, creatinine, FBG, HbA1c, fibrinogen, and D-dimer concentrations. Lipid profile parameters included triglycerides, total cholesterol, LDL-C, and HDL-C. Cardiac biomarkers and echocardiographic indices included NT-proBNP levels and LVEF. Medication use was also recorded, including antihypertensive drugs, antidiabetic agents, lipid-lowering drugs, β-blockers, diuretics, and anticoagulants. All variables were predefined and systematically collected to ensure data completeness and the reproducibility of analyses.

### 2.4 Follow-up and study endpoints

Follow-up for this study began at the date of hospital discharge, and patients were monitored until death or July 2025, whichever occurred first. Follow-up data were obtained through outpatient and emergency clinic visits, review of medical records, and structured telephone interviews. All enrolled patients completed follow-up until death or the predefined study end date (July 2025). Therefore, no attrition bias due to incomplete follow-up occurred in the present study. The MACE was defined as a composite of the following outcomes: (1) all-cause death; (2) cardiovascular death; (3) non-fatal MI; (4) non-fatal stroke; (5) unplanned coronary revascularization; and (6) hospitalization for worsening HF. Based on the occurrence of MACE during follow-up (median duration: 35 months), patients were stratified into two groups: the non-MACE group (*n* = 603) and the MACE group (*n* = 131).

### 2.5 Statistical analysis

All statistical analyses were performed using SPSS software, version 28.0 (IBM Corp., Armonk, NY, United States) and R software, version 4.4.2 (R Foundation for Statistical Computing, Vienna, Austria). Because all variables included in the statistical analyses were complete, no multiple imputation or other missing data handling procedures were performed. Normality of continuous variables was examined with the Shapiro–Wilk test. Variables with a non-normal distribution were summarized as medians with interquartile ranges and compared across groups using the Kruskal–Wallis test. Categorical variables were expressed as counts and percentages, and differences between groups were assessed with the chi-square test. In outcome analysis, the primary endpoint was the occurrence of MACE. All-cause mortality was also analyzed as a secondary outcome. Kaplan–Meier survival curves were generated to estimate event-free survival across different PNI groups, and comparisons were performed with the log-rank test.

The relationship between PNI and the risk of MACE and all-cause mortality was further evaluated using the Cox proportional hazards regression model, where PNI was analyzed both as a continuous variable (original and standardized values) and as a categorical variable (based on the median, optimal cutoff, tertiles, and quartiles). For MACE, three Cox regression models were constructed: Model 1 adjusted for age; Model 2 additionally adjusted for smoking status, NYHA class III–IV, hypertension, CHD, stroke, CKD, and the CHA_2_DS_2_-VASc score; and Model 3 further adjusted for heart rate, total cholesterol, HDL-C, FBG, fibrinogen, LVEF, use of β-blockers, and anticoagulants. For all-cause mortality, Model 1 was unadjusted; Model 2 adjusted for NYHA class III–IV, hemoglobin, and total cholesterol; and Model 3 further adjusted for NT-proBNP and use of β-blockers. HRs with 95% confidence intervals (CIs) were reported. All covariates included in these models were selected based on their statistically significant association with the outcomes in univariate analyses (*P* < 0.05). To evaluate potential multicollinearity among covariates included in the multivariable models, variance inflation factors (VIFs) were calculated for all independent variables. Serum albumin and lymphocyte count were not entered separately into the regression models to avoid structural collinearity with PNI, which is mathematically derived from these two components. For the remaining covariates (including NT-proBNP, renal function indices, inflammatory markers, and clinical severity indicators), VIF values were examined, and all were below commonly accepted thresholds (VIF < 5), indicating no evidence of significant multicollinearity.

To examine the robustness of findings, subgroup analyses were conducted across strata including age, gender, NYHA class, hypertension, diabetes, dyslipidemia, CHD, stroke, and CKD. The association between PNI (as either a continuous or categorical variable) and MACE was assessed within each subgroup. Given the multiple subgroup and threshold-based analyses performed, these exploratory analyses were primarily conducted to evaluate the consistency and robustness of associations rather than to develop a predictive model. Formal correction for multiple comparisons was not applied; therefore, subgroup findings should be interpreted as hypothesis-generating. To mitigate potential reverse causality, a sensitivity analysis excluding patients who died within the first six months of follow-up was additionally performed. Additionally, the ROC curve and the area under the curve (AUC) were applied to evaluate the discriminatory performance of PNI for MACE and all-cause mortality. The optimal cutoff value of PNI was determined using the Youden index derived from the ROC curve within the study cohort. Because this threshold was generated from the same dataset and was not subjected to internal cross-validation or external validation, it should be considered exploratory in nature. To further explore potential dose–response relationships, a restricted cubic spline (RCS) model was used in multivariable-adjusted analyses to assess potential non-linear associations between PNI with MACE and all-cause mortality. The RCS model was constructed using three knots placed at the 10th, 50th, and 90th percentiles of the PNI distribution. Non-linearity was formally tested using a likelihood ratio test comparing the model containing spline terms with a model including only the linear term. A non-significant *P*-value for non-linearity was interpreted as evidence supporting a linear association. A *P*< 0.05 (two-tailed) was considered to indicate statistical significance.

## 3 Results

### 3.1 Baseline clinical characteristics

Among the 734 enrolled patients, individuals were stratified into three groups according to PNI tertiles (T1, T2, and T3). Several baseline characteristics differed significantly across groups (Table 1), including age, NYHA functional class, BMI, selected hematological and biochemical parameters, NT-proBNP levels, and certain medication use (all *P* < 0.05). More importantly, significant differences were observed in clinical outcomes across PNI tertiles. The incidence of MACE decreased progressively with increasing PNI (27.2% in T1, 16.8% in T2, and 9.4% in T3; P < 0.001). Similarly, all-cause mortality was highest in the lowest PNI group and declined across tertiles (8.1% in T1, 4.1% in T2, and 1.2% in T3; P = 0.001).

**TABLE 1:** Clinical characteristics grouped by PNI tertiles.

Variables	All patients	T1	T2	T3	*P*-value
N	734	246	244	244	< 0.001
Age, years	77.00 (70.00, 84.00)	79.00 (71.00, 85.00)	79.00 (70.00, 85.00)	75.00 (68.00, 82.00)	
Male, n (%)	334 (45.5%)	116 (47.2%)	113 (46.3%)	105 (43.0%)	0.626
Smoking, n (%)	137 (18.7%)	36 (14.6%)	56 (23.0%)	45 (18.4%)	0.061
NYHA class, n (%)					0.004
I-II	298 (40.6%)	79 (32.1%)	112 (45.9%)	107 (43.9%)	
III-IV	436 (59.4%)	167 (67.9%)	132 (54.1%)	137 (56.1%)	
Hypertension, n (%)	408 (55.6%)	140 (56.9%)	134 (54.9%)	134 (54.9%)	0.877
Diabetes, n (%)	347 (47.3%)	110 (44.7%)	110 (45.1%)	127 (52.0%)	0.187
Dyslipidemia, n (%)	292 (39.8%)	101 (41.1%)	84 (34.4%)	107 (43.9%)	0.092
CHD, n (%)	139 (18.9%)	52 (21.1%)	49 (20.1%)	38 (15.6%)	0.249
Stroke, n (%)	115 (15.7%)	45 (18.3%)	43 (17.6%)	27 (11.1%)	0.052
CKD, n (%)	179 (24.4%)	63 (25.6%)	69 (28.3%)	47 (19.3%)	0.058
CHA_2_DS_2_-VASc score	4.00 (2.00, 5.00)	4.00 (3.00, 5.00)	4.00 (3.00, 5.00)	3.00 (2.00, 4.00)	0.136
BMI, kg/m^2^	24.34 (20.50, 27.89)	22.84 (19.84, 27.34)	24.22 (20.54, 27.61)	25.46 (21.37, 28.87)	0.001
Heart rate, beats/minute	86.00 (74.00, 101.00)	89.00 (75.00, 102.00)	84.00 (74.00, 101.50)	85.00 (73.50, 98.50)	0.081
Systolic blood pressure, mmHg	131.00 (116.00, 150.00)	131.50 (117.00, 148.00)	130.00 (115.00, 147.50)	133.50 (116.00, 153.00)	0.403
Diastolic blood pressure, mmHg	78.00 (68.00, 87.00)	78.50 (68.00, 89.00)	78.00 (66.00, 86.50)	78.00 (68.00, 87.00)	0.714
WBC, ×10^9^/L	6.92 (5.64, 8.87)	6.60 (5.12, 8.54)	6.76 (5.36, 8.68)	7.38 (6.24, 9.23)	< 0.001
Neutrophil count, ×10^9^/L	4.95 (3.71, 6.75)	5.09 (3.45, 7.00)	4.93 (3.64, 6.76)	4.95 (3.97, 6.57)	0.846
Lymphocyte count, ×10^9^/L	1.19 (0.83, 1.61)	0.85 (0.64, 1.10)	1.13 (0.88, 1.48)	1.68 (1.29, 2.21)	< 0.001
Monocyte count, ×10^9^/L	0.46 (0.33, 0.62)	0.46 (0.31, 0.59)	0.46 (0.32, 0.64)	0.48 (0.36, 0.65)	0.035
Hemoglobin, g/L	129.00 (116.00, 141.00)	124.00 (108.00, 135.00)	128.00 (115.00, 140.00)	135.00 (125.00, 146.00)	< 0.001
Platelet count, ×10^9^/L	170.00 (133.00, 220.00)	154.50 (125.00, 209.00)	170.00 (133.50, 225.00)	185.00 (143.00, 224.00)	< 0.001
Albumin, g/L	38.39 (35.95, 40.90)	35.35 (33.53, 36.80)	38.89 (37.24, 40.34)	41.73 (39.80, 43.60)	< 0.001
Uric acid, μmol/L	366.50 (287.00, 480.00)	366.50 (291.00, 486.00)	380.50 (275.00, 483.00)	361.00 (292.50, 477.50)	0.895
Creatinine, μmol/L	77.00 (62.00, 101.00)	79.00 (63.75, 105.00)	80.00 (63.00, 104.75)	73.00 (59.25, 94.00)	0.037
Triglyceride, mmol/L	1.16 (0.84, 1.60)	1.04 (0.74, 1.40)	1.07 (0.82, 1.57)	1.35 (1.01, 2.01)	< 0.001
Total cholesterol, mmol/L	3.96 (3.23, 4.74)	3.64 (2.93, 4.33)	4.03 (3.30, 4.75)	4.23 (3.48, 5.08)	< 0.001
LDL-C, mmol/L	2.13 (1.67, 2.61)	1.98 (1.53, 2.46)	2.17 (1.70, 2.60)	2.24 (1.80, 2.85)	< 0.001
HDL-C, mmol/L	1.20 (0.96, 1.47)	1.15 (0.95, 1.42)	1.20 (0.98, 1.50)	1.25 (0.98, 1.50)	0.025
Fasting blood glucose, mmol/L	6.13 (5.40, 6.70)	6.00 (5.24, 6.52)	6.11 (5.40, 6.69)	6.33 (5.40, 7.11)	0.029
HbA1c, (%)	6.10 (5.70, 6.70)	6.10 (5.70, 6.50)	5.95 (5.60, 6.70)	6.20 (5.70, 6.80)	0.125
Fibrinogen, g/L	3.03 (2.55, 3.58)	3.17 (2.54, 3.84)	3.17 (2.67, 3.71)	2.86 (2.53, 3.32)	0.002
D-dimer, mg/L	1.02 (0.50, 2.10)	1.32 (0.76, 2.34)	0.87 (0.42, 2.03)	0.83 (0.37, 1.66)	< 0.001
NT-proBNP, pg/mL	4,054.50 (2,263.00, 8,245.60)	5,499.50 (2,653.00, 10,721.00)	3,801.75 (2,195.00, 7,948.00)	3,687.35 (1,984.50, 6,524.00)	< 0.001
LVEF, %	68.00 (65.00, 70.00)	68.00 (65.00, 70.00)	67.00 (64.00, 70.00)	68.00 (65.00, 71.50)	0.024
Antihypertensive drugs, n (%)	334 (45.5%)	96 (39.0%)	120 (49.2%)	118 (48.4%)	0.043
Antidiabetic drugs, n (%)	219 (29.8%)	71 (28.9%)	63 (25.8%)	85 (34.8%)	0.086
Lipid-lowering drugs, n (%)	139 (18.9%)	33 (13.4%)	39 (16.0%)	67 (27.5%)	< 0.001
β-blockers, n (%)	442 (60.2%)	142 (57.7%)	148 (60.7%)	152 (62.3%)	0.578
Diuretics, n (%)	276 (37.6%)	94 (38.2%)	97 (39.8%)	85 (34.8%)	0.518
Anticoagulants, n (%)	536 (73.0%)	199 (80.9%)	170 (69.7%)	167 (68.4%)	0.003
MACE, n (%)					< 0.001
No	603 (82.2%)	179 (72.8%)	203 (83.2%)	221 (90.6%)	
Yes	131 (17.8%)	67 (27.2%)	41 (16.8%)	23 (9.4%)	
All-cause mortality, n (%)					0.001
No	701 (95.5%)	226 (91.9%)	234 (95.9%)	241 (98.8%)	
Yes	33 (4.5%)	20 (8.1%)	10 (4.1%)	3 (1.2%)	

PNI, prognostic nutritional index; CHD, coronary heart disease; CKD, chronic kidney disease; BMI, body mass index; WBC, white blood cell count; LDL-C, low-density lipoprotein cholesterol; HDL-C, high-density lipoprotein cholesterol; HbA1c, glycated hemoglobin; NT-proBNP, N-terminal pro-brain natriuretic peptide; LVEF, left ventricular ejection fraction; MACE, major adverse cardiovascular events.

### 3.2 Association between PNI and MACE and all-cause mortality

**FIGURE 1:**
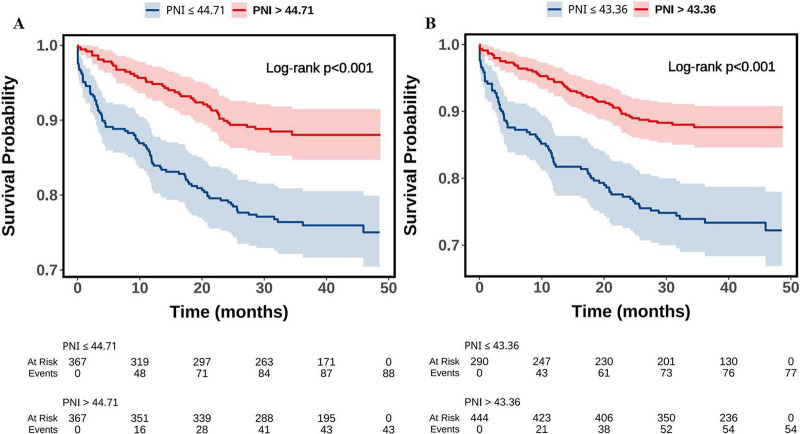
Kaplan-Meier survival curves of MACE-free survival probability grouped by PNI median **(A)** and optimal cutoff value **(B)**. PNI, prognostic nutritional index; MACE, major adverse cardiovascular events.

**FIGURE 2:**
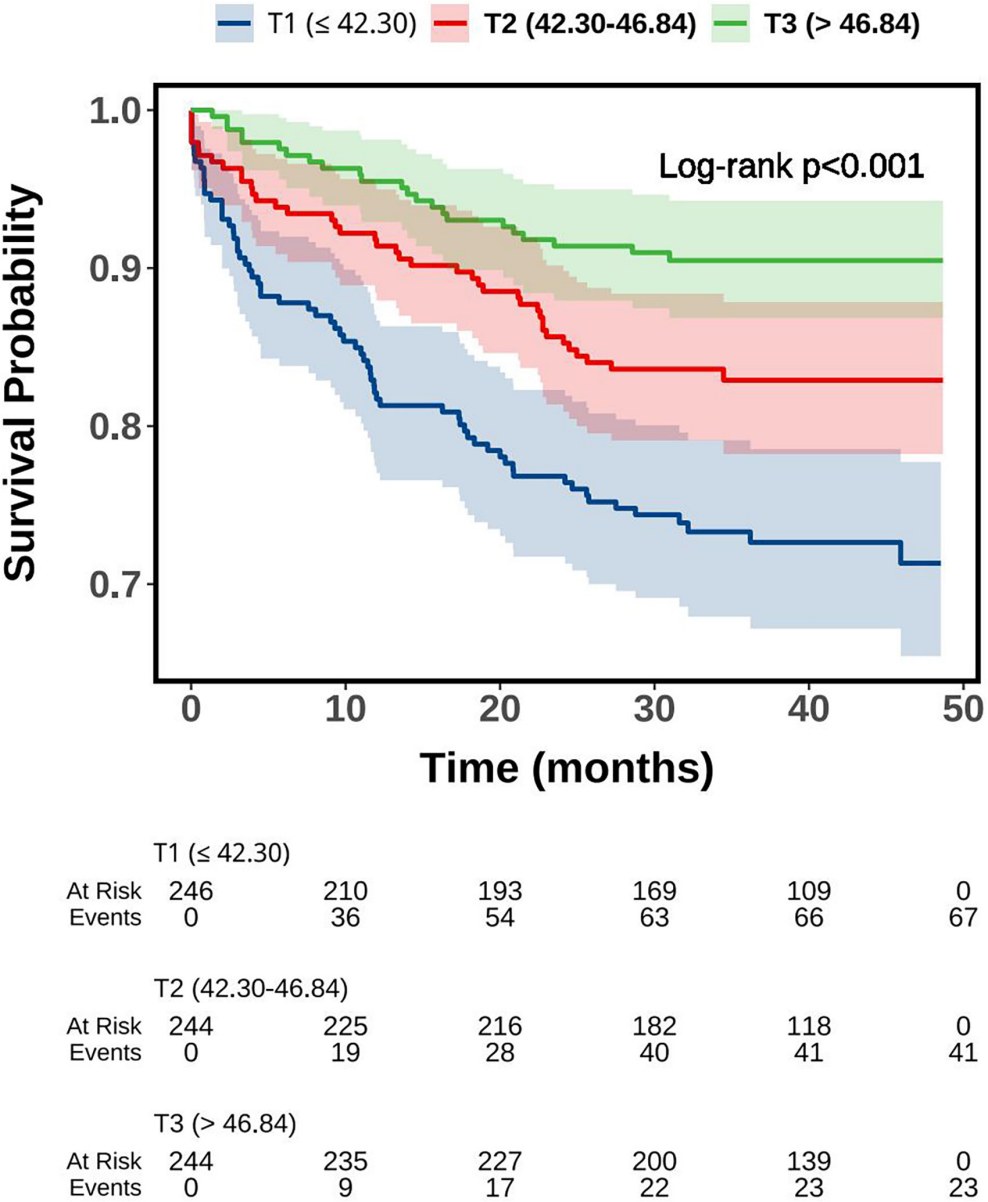
Kaplan-Meier survival curve of MACE-free survival probability grouped by PNI tertiles. PNI, prognostic nutritional index; MACE, major adverse cardiovascular events.

**FIGURE 3:**
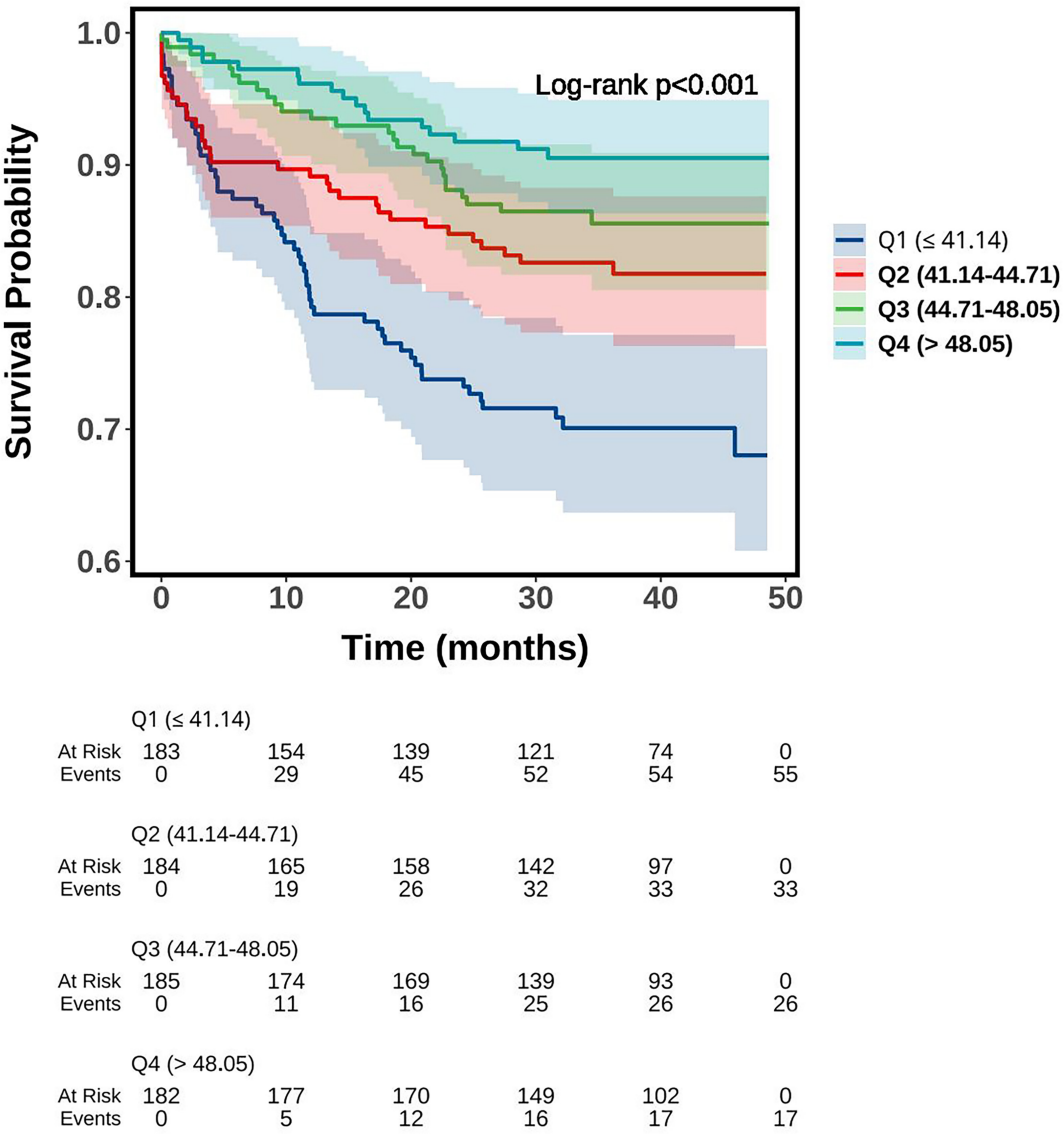
Kaplan-Meier survival curve of MACE-free survival probability grouped by PNI quartiles. PNI, prognostic nutritional index; MACE, major adverse cardiovascular events.

Kaplan–Meier survival analysis demonstrated significant differences in MACE-free survival across different PNI categories (all log-rank tests, *P* < 0.001). When stratified by the median cutoff (44.71) and the optimal threshold (43.36), patients with lower PNI values (PNI ≤ 44.71 or PNI ≤ 43.36) exhibited a markedly higher cumulative incidence of MACE compared with those in the higher PNI groups (Figures 1A,B). Further stratification by tertiles revealed a stepwise trend: patients in the T1 group (PNI ≤ 42.30) showed the highest incidence of MACE, whereas survival curves in T2 (42.30 < PNI ≤ 46.84) and T3 (PNI > 46.84) progressively shifted upward, reflecting a gradual reduction in risk (Figure 2). A similar pattern was observed in the quartile-based analysis, where individuals in Q1 (PNI ≤ 41.14) experienced the poorest outcomes, while prognosis improved sequentially in Q2 (41.14 < PNI ≤ 44.71), Q3 (44.71 < PNI ≤ 48.05), and Q4 (PNI > 48.05), indicating a clear dose–response association between higher PNI and reduced MACE risk (Figure 3).

The multivariable Cox regression analysis demonstrated that both continuous and categorical forms of the PNI were significantly and inversely associated with the occurrence of MACE (Table 2). (1) When PNI was analyzed as a continuous variable, the HRs were 0.897 (95% CI: 0.864–0.932) in Model 1 and 0.899 (95% CI: 0.865–0.934) in Model 2. After further adjustment for additional clinical and laboratory covariates in Model 3, the association remained robust with an HR of 0.904 (95% CI: 0.869–0.940, *P* < 0.001). Similarly, standardized PNI consistently showed a negative association with MACE across all models, with HRs of 0.556, 0.561, and 0.578, respectively (all *P* < 0.001). (2) When PNI was evaluated as a categorical variable, using the lowest tertile (T1, PNI ≤ 42.30) as the reference, the HRs for the middle tertile (T2, 42.30 < PNI ≤ 46.84) were 0.574 (95% CI: 0.389–0.847), 0.544 (95% CI: 0.368–0.806), and 0.556 (95% CI: 0.374–0.826) across the three models. For the highest tertile (T3, PNI > 46.84), the HRs were 0.307 (95% CI: 0.191–0.494), 0.319 (95% CI: 0.198–0.513), and 0.366 (95% CI: 0.226–0.591), respectively.

**TABLE 2:** The multivariate association between PNI with MACE and all-cause mortality.

Variables	Model 1			Model 2			Model 3		
	HR	95% CI	*P* value	HR	95% CI	*P* value	HR	95% CI	P value
MACE									
As a continuous variable									
PNI	0.897	0.864–0.932	< 0.001	0.899	0.865–0.934	< 0.001	0.904	0.869–0.940	< 0.001
Standardized PNI	0.556	0.453–0.682	< 0.001	0.561	0.457–0.689	< 0.001	0.578	0.467–0.716	< 0.001
As a categorical variable									
T1	Reference			Reference			Reference		
T2	0.574	0.389–0.847	0.005	0.544	0.368–0.806	0.002	0.556	0.374–0.826	0.004
T3	0.307	0.191-0.494	< 0.001	0.319	0.198–0.513	< 0.001	0.366	0.226–0.591	< 0.001
P for trend			< 0.001			< 0.001			< 0.001
All-cause mortality									
As a continuous variable									
PNI	0.837	0.773–0.907	< 0.001	0.853	0.788–0.922	< 0.001	0.851	0.785–0.922	< 0.001
Standardized PNI	0.382	0.247–0.591	< 0.001	0.422	0.276–0.644	< 0.001	0.417	0.270–0.645	< 0.001
As a categorical variable									
T1	Reference			Reference			Reference		
T2	0.498	0.233–1.063	0.072	0.580	0.270–1.242	0.160	0.594	0.277-1.273	0.181
T3	0.146	0.044–0.493	0.002	0.165	0.049-0.556	0.004	0.174	0.052-0.588	0.005
P for trend			0.001			0.002			0.003

For MACE, Model 1 adjusted for age only; Model 2 adjusted for age, smoking, NYHA class III–IV, hypertension, coronary heart disease, stroke, chronic kidney disease, and CHA_2_DS_2_-VASc score; Model 3 adjusted for age, smoking, NYHA class III–IV, hypertension, coronary heart disease, stroke, chronic kidney disease, CHA_2_DS_2_-VASc score, heart rate, total cholesterol, high-density lipoprotein cholesterol, fasting blood glucose, fibrinogen, left ventricular ejection fraction, β-blockers, and anticoagulants. For all-cause mortality, Model 1 did not adjust for covariates; Model 2 adjusted for NYHA class III–IV, hemoglobin, and total cholesterol; Model 3 adjusted for NYHA class III–IV, hemoglobin, total cholesterol, N-terminal pro-brain natriuretic peptide, and β-blockers. MACE, major adverse cardiovascular events; PNI, prognostic nutritional index; CI, confidence interval; HR, hazard ratio.

**FIGURE 4:**
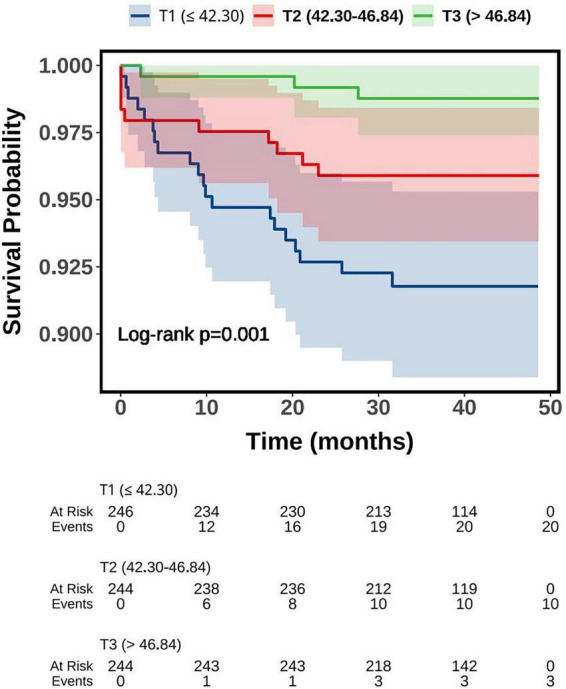
Kaplan-Meier survival curve of all-cause mortality-free survival probability grouped by PNI tertiles. PNI, prognostic nutritional index.

In addition to MACE, as shown in Table 2, PNI was also significantly and inversely associated with the risk of death. In the fully adjusted model (Model 3), each unit increase in PNI was associated with a 14.9% reduction in mortality risk (HR = 0.851, 95% CI: 0.785–0.922, *P* < 0.001). The standardized PNI also showed strong predictive value, with an HR of 0.417 (95% CI: 0.270–0.645, *P* < 0.001). When analyzed categorically, patients in the highest tertile (T3) had a significantly lower risk of all-cause mortality compared to those in T1, with HRs of 0.146 (95% CI: 0.044–0.493, *P* = 0.002), 0.165 (95% CI: 0.049–0.556, *P* = 0.004), and 0.174 (95% CI: 0.052–0.588, P = 0.005) across Models 1–3. Furthermore, Kaplan–Meier survival curves for all-cause mortality across PNI tertiles further supported these findings (Figure 4). Patients in the lowest PNI tertile (T1) had the poorest survival, while those in the highest tertile (T3) demonstrated the best outcomes, with statistically significant differences between the three groups (log-rank *P* = 0.001).

### 3.3 Subgroup-specific associations between PNI and MACE

Subgroup analyses demonstrated that PNI was significantly and inversely associated with the risk of MACE across most clinical strata (Table 3). In patients over 75 years, each 1-unit and 1-SD increase in PNI was associated with a 16.3% and 61.9% lower risk of MACE (HR = 0.837 and 0.381). In males, the corresponding reductions were 13.2% and 53.6% (HR = 0.868 and 0.464), while in females they were 9.1% and 40.5% (HR = 0.909 and 0.595). A similar trend was observed across HF severity: in NYHA class III–IV, risk decreased by 10.2 and 44.0% (HR = 0.898 and 0.560), whereas in class I–II the reductions were smaller at 8.0 and 36.5%. In hypertensive patients, PNI increments of 1 unit and 1 SD were associated with 10.7 and 45.7% (HR = 0.893 and 0.543) lower risk, while in non-hypertensive individuals, reductions were 9.0 and 40.1% (HR = 0.910 and 0.599). In diabetes patients, risk fell by 11.1 and 47.2% (HR = 0.889 and 0.528), compared with 7.1 and 32.9% (HR = 0.929 and 0.671) in non-diabetics. In dyslipidemic patients, reductions reached 9.2 and 40.9% (HR = 0.908 and 0.591), whereas in those without dyslipidemia, the declines were 10.7 and 45.8% (HR = 0.893 and 0.542). In patients with CHD, risk reductions were more modest at 6.2 and 29.4% (HR = 0.938 and 0.706), but in those without CHD, the decreases were 11.4 and 48.0% (HR = 0.886 and 0.520). For patients without stroke, risk fell by 9.3 and 41.0% (HR = 0.907 and 0.590). In CKD patients, PNI increments were associated with 10.8% and 46.0% (HR = 0.892 and 0.540) lower risk, and in non-CKD patients, the corresponding reductions were 9.1 and 40.4% (HR = 0.909 and 0.596).

**TABLE 3:** Subgroup association between PNI as a continuous variable and MACE.

Subgroups	PNI		Standardized PNI	
	HR (95% CI)	*P-*value	HR (95% CI)	*P*-value
Age				
≤ 75 years	0.952 (0.898, 1.009)	0.099	0.766 (0.558, 1.051)	0.099
> 75 years	0.837 (0.788, 0.889)	< 0.001	0.381 (0.275, 0.528)	< 0.001
Gender				
Male	0.868 (0.816, 0.923)	< 0.001	0.464 (0.332, 0.647)	< 0.001
Female	0.909 (0.862, 0.958)	< 0.001	0.595 (0.447, 0.792)	< 0.001
NYHA class				
I-II	0.920 (0.860, 0.983)	0.014	0.635 (0.442, 0.913)	0.014
III-IV	0.898 (0.856, 0.943)	< 0.001	0.560 (0.430, 0.729)	< 0.001
Hypertension				
Yes	0.893 (0.849, 0.940)	< 0.001	0.543 (0.413, 0.714)	< 0.001
No	0.910 (0.855, 0.969)	0.003	0.599 (0.427, 0.842)	0.003
Diabetes				
Yes	0.889 (0.841, 0.940)	< 0.001	0.528 (0.391, 0.713)	< 0.001
No	0.929 (0.879, 0.982)	0.010	0.671 (0.497, 0.908)	0.010
Dyslipidemia				
Yes	0.908 (0.858, 0.960)	0.001	0.591 (0.436, 0.802)	0.001
No	0.893 (0.846, 0.943)	< 0.001	0.542 (0.405, 0.726)	< 0.001
Coronary heart disease				
Yes	0.938 (0.880, 0.999)	0.048	0.706 (0.500, 0.996)	0.048
No	0.886 (0.842, 0.933)	< 0.001	0.520 (0.394, 0.686)	< 0.001
Stroke				
Yes	0.922 (0.829, 1.025)	0.134	0.643 (0.361, 1.146)	0.134
No	0.907 (0.869, 0.947)	< 0.001	0.590 (0.467, 0.746)	< 0.001
Chronic kidney disease				
Yes	0.892 (0.817, 0.975)	0.011	0.540 (0.335, 0.870)	0.011
No	0.909 (0.869, 0.951)	< 0.001	0.596 (0.466, 0.761)	< 0.001

Subgroup analyses adjusted for age, smoking, NYHA class III–IV, hypertension, coronary heart disease, stroke, chronic kidney disease, CHA_2_DS_2_-VASc score, heart rate, total cholesterol, high-density lipoprotein cholesterol, fasting blood glucose, fibrinogen, left ventricular ejection fraction, β-blockers, and anticoagulants. MACE, major adverse cardiovascular events; PNI, prognostic nutritional index; CI, confidence interval; HR, hazard ratio.

When PNI was analyzed as a categorical variable, using the T1 group as the reference, most subgroups demonstrated a significant reduction in the risk of MACE among patients in T2 and T3 (Table 4). Formal interaction testing showed no statistically significant interaction in most subgroups (all P for interaction > 0.05), suggesting no strong evidence of effect modification. However, a significant interaction was observed for age (P for interaction < 0.001). In patients over 75 years, the risk decreased by 48.9 and 89.2% in T2 and T3, respectively (HR = 0.511 and 0.108). In men, the T3 group showed a 59.5% risk reduction (HR = 0.405), while in women, the reductions were 41.8 and 64.0% in T2 and T3 (HR = 0.582 and 0.360). For patients with NYHA class III–IV, T2 and T3 were associated with 55.7 and 61.0% lower risks (HR = 0.443 and 0.390). In those with hypertension, the risk declined by 42.1 and 76.1% in T2 and T3 (HR = 0.579 and 0.239). Among diabetic patients, the reductions were 51.8 and 63.9% (HR = 0.482 and 0.361), while in non-diabetic patients, the T3 group showed a 59.8% decrease (HR = 0.402). In individuals without dyslipidemia, T2 and T3 were associated with 47.5 and 64.2% lower risks (HR = 0.525 and 0.358), whereas in those with dyslipidemia, the T3 group showed a 68.6% reduction (HR = 0.314). For patients with CHD, the T2 group showed a 57.8% reduction (HR = 0.422), and in those without CHD, the T3 group showed a 70.3% decrease (HR = 0.297). In patients without prior stroke, T2 and T3 were associated with 36.8 and 63.6% lower risks (HR = 0.632 and 0.364). Finally, among patients with CKD, the risk declined by 53.9 and 72.7% in T2 and T3 (HR = 0.461 and 0.273), while in those without CKD, the T3 group showed a 59.9% reduction (HR = 0.401).

**TABLE 4:** Subgroup association between PNI as a categorical variable and MACE.

Subgroups	T2 vs. T1		T3 vs. T1			
	HR (95% CI)	*P*-value	HR (95% CI)	*P*-value	P for trend	P for interaction
Age						< 0.001
≤ 75 years	0.523 (0.262, 1.045)	0.067	0.732 (0.363, 1.479)	0.385	0.184	
> 75 years	0.511 (0.311, 0.842)	0.008	0.108 (0.039, 0.304)	< 0.001	< 0.001	
Gender						0.664
Male	0.576 (0.311, 1.066)	0.079	0.405 (0.194, 0.842)	0.015	0.030	
Female	0.582 (0.348, 0.974)	0.039	0.360 (0.188, 0.688)	0.002	0.003	
NYHA class						0.531
I-II	0.961 (0.462, 1.998)	0.915	0.442 (0.170, 1.151)	0.095	0.182	
III-IV	0.443 (0.268, 0.734)	0.002	0.390 (0.217, 0.701)	0.002	< 0.001	
Hypertension						0.054
Yes	0.579 (0.357, 0.940)	0.027	0.239 (0.122, 0.467)	< 0.001	< 0.001	
No	0.573 (0.276, 1.193)	0.137	0.685 (0.309, 1.518)	0.351	0.306	
Diabetes						0.719
Yes	0.482 (0.271, 0.858)	0.013	0.361 (0.190, 0.688)	0.002	0.002	
No	0.652 (0.380, 1.118)	0.120	0.402 (0.194, 0.832)	0.014	0.029	
Dyslipidemia						0.423
Yes	0.716 (0.394, 1.301)	0.272	0.314 (0.152, 0.648)	0.002	0.003	
No	0.525 (0.314, 0.880)	0.014	0.358 (0.189, 0.677)	0.002	0.002	
Coronary heart disease						0.070
Yes	0.422 (0.218, 0.817)	0.010	0.677 (0.333, 1.375)	0.280	0.031	
No	0.640 (0.387, 1.059)	0.082	0.297 (0.150, 0.592)	0.001	0.001	
Stroke						0.322
Yes	0.426 (0.166, 1.094)	0.076	0.628 (0.202, 1.951)	0.422	0.198	
No	0.632 (0.403, 0.991)	0.046	0.364 (0.210, 0.631)	< 0.001	0.001	
Chronic kidney disease						0.893
Yes	0.461 (0.223, 0.954)	0.037	0.273 (0.076, 0.973)	0.045	0.032	
No	0.663 (0.408, 1.076)	0.096	0.401 (0.234, 0.687)	0.001	0.002	

Subgroup analyses adjusted for age, smoking, NYHA class III–IV, hypertension, coronary heart disease, stroke, chronic kidney disease, CHA_2_DS_2_-VASc score, heart rate, total cholesterol, high-density lipoprotein cholesterol, fasting blood glucose, fibrinogen, left ventricular ejection fraction, β-blockers, and anticoagulants. PNI, prognostic nutritional index; MACE, major adverse cardiovascular events; HR, hazard ratio; CI, confidence interval.

### 3.4 Association between PNI and MACE and all-cause mortality based on different grouping thresholds

The relationship between PNI and MACE was further examined using multiple stratification approaches (Table 5). When patients were divided according to the median PNI value of 44.71, those in the higher PNI group consistently demonstrated a significantly lower risk of MACE across all three models, with HRs of 0.446 (95% CI: 0.310–0.643), 0.446 (95% CI: 0.309–0.644), and 0.506 (95% CI: 0.349–0.735) (all *P* < 0.001). A similar pattern was observed when stratification was performed using the optimal cutoff value of 43.36, where higher PNI was again associated with reduced risk, yielding HRs of 0.414 (95% CI: 0.292–0.586), 0.402 (95% CI: 0.283–0.572), and 0.469 (95% CI: 0.328–0.672) (all *P* < 0.001). In the quartile-based analysis, using Q1 as the reference, patients in Q2, Q3, and Q4 showed progressively lower risks of MACE. In Model 3, the HRs were 0.595 (95% CI: 0.384–0.921, *P* = 0.020), 0.444 (95% CI: 0.277–0.713, *P* = 0.001), and 0.325 (95% CI: 0.187–0.565, *P* < 0.001), respectively.

**TABLE 5:** The association between PNI grouped by different thresholds and MACE and all-cause mortality.

Variables	Model 1			Model 2			Model 3		
	HR	95% CI	*P*-value	HR	95% CI	*P*-value	HR	95% CI	*P*-value
MACE									
Grouped by median (44.71)									
Low PNI	Reference			Reference			Reference		
High PNI	0.446	0.310–0.643	< 0.001	0.446	0.309–0.644	< 0.001	0.506	0.349–0.735	< 0.001
Grouped by optimal cutoff value (43.36)									
Low PNI	Reference			Reference			Reference		
High PNI	0.414	0.292–0.586	< 0.001	0.402	0.283–0.572	< 0.001	0.469	0.328–0.672	< 0.001
Grouped by quartiles									
Q1	Reference			Reference			Reference		
Q2	0.554	0.360–0.853	0.007	0.599	0.388–0.923	0.020	0.595	0.384–0.921	0.020
Q3	0.416	0.261–0.663	< 0.001	0.402	0.251–0.641	< 0.001	0.444	0.277–0.713	0.001
Q4	0.270	0.157–0.465	< 0.001	0.288	0.167–0.498	< 0.001	0.325	0.187–0.565	< 0.001
P for trend			< 0.001			< 0.003			< 0.001
All-cause mortality									
Grouped by median (44.71)									
Low PNI	Reference	–		Reference			Reference		
High PNI	0.216	0.089–0.524	< 0.001	0.270	0.110–0.661	0.004	0.270	0.110–0.661	0.004
Grouped by optimal cutoff value (42.95)									
Low PNI	Reference			Reference			Reference		
High PNI	0.222	0.103–0.478	< 0.001	0.248	0.115–0.535	< 0.001	0.258	0.120–0.557	0.001
Grouped by quartiles									
Q1	Reference			Reference			Reference		
Q2	0.580	0.266–1.267	0.172	0.673	0.307–1.473	0.321	0.673	0.307–1.474	0.322
Q3	0.226	0.076–0.673	0.007	0.262	0.088–0.779	0.016	0.284	0.095–0.848	0.024
Q4	0.114	0.026–0.494	0.004	0.130	0.030–0.564	0.006	0.134	0.031–0.580	0.007
P for trend			0.001			0.002			0.003

For MACE, Model 1 adjusted for age only; Model 2 adjusted for age, smoking, NYHA class III–IV, hypertension, coronary heart disease, stroke, chronic kidney disease, and CHA_2_DS_2_-VASc score; Model 3 adjusted for age, smoking, NYHA class III–IV, hypertension, coronary heart disease, stroke, chronic kidney disease, CHA_2_DS_2_-VASc score, heart rate, total cholesterol, high-density lipoprotein cholesterol, fasting blood glucose, fibrinogen, left ventricular ejection fraction, β-blockers, and anticoagulants. For all-cause mortality, Model 1 did not adjust for covariates; Model 2 adjusted for NYHA class III–IV, hemoglobin, and total cholesterol; Model 3 adjusted for NYHA class III–IV, hemoglobin, total cholesterol, N-terminal pro-brain natriuretic peptide, and β-blockers. PNI, prognostic nutritional index; MACE, major adverse cardiovascular events; HR, hazard ratio; CI, confidence interval.

Consistent results were observed when analyzing all-cause mortality across the same PNI grouping strategies. When dichotomized by the median (44.71), the higher PNI group showed significantly reduced risk of death, with HRs of 0.216 (95% CI: 0.089–0.524), 0.270 (95% CI: 0.110–0.661), and 0.270 (95% CI: 0.110–0.661) in Models 1–3 (all *P* < 0.01). Using the optimal cutoff value (42.95), higher PNI remained protective, with HRs of 0.222 (95% CI: 0.103–0.478), 0.248 (95% CI: 0.115–0.535), and 0.258 (95% CI: 0.120–0.557), all highly significant (*P* ≤ 0.001). In the quartile-based analysis, a clear inverse dose–response trend was again evident (P for trend < 0.01 in all models). Compared with Q1, patients in Q3 and Q4 had substantially lower mortality risks. In Model 3, the HRs were 0.284 (95% CI: 0.095–0.848, *P* = 0.024) for Q3 and 0.134 (95% CI: 0.031–0.580, *P* = 0.007) for Q4.

To address the potential influence of reverse causality, a sensitivity analysis was conducted by excluding patients who died within the first 6 months of follow-up. After removing these early events, the multivariable Cox regression analyses were repeated (Table 6). The association between PNI and MACE remained stable across all three models. In the fully adjusted Model 3, PNI as a continuous variable was still significantly associated with a lower risk of MACE (HR = 0.911, 95% CI: 0.875–0.949, *P* < 0.001). Similarly, when analyzed as tertiles, both T2 and T3 were associated with significantly lower risks of MACE compared with T1 (HR = 0.525 and 0.377, respectively; both *P* < 0.01), and the trend test remained significant (P for trend < 0.001). For all-cause mortality, PNI as a continuous variable also remained significantly associated with reduced mortality risk across the models (Model 3: HR = 0.847, 95% CI: 0.763–0.941, *P* = 0.002).

**TABLE 6:** The multivariate association between PNI with MACE and all-cause mortality: exclude patients who died within 6 months.

Variables	Model 1			Model 2			Model 3		
	HR	95% CI	*P*-value	HR	95% CI	*P*-value	HR	95% CI	*P*-value
MACE									
As a continuous variable									
PNI	0.906	0.871-0.942	< 0.001	0.906	0.871–0.943	< 0.001	0.911	0.875–0.949	< 0.001
Standardized PNI	0.585	0.472–0.724	< 0.001	0.586	0.472–0.728	< 0.001	0.604	0.484–0.753	< 0.001
As a categorical variable									
T1	Reference			Reference			Reference		
T2	0.566	0.374–0.857	0.007	0.525	0.345–0.798	0.003	0.525	0.344–0.800	0.003
T3	0.329	0.202–0.537	< 0.001	0.338	0.207–0.552	< 0.001	0.377	0.230–0.619	< 0.001
P for trend			< 0.001			< 0.001			< 0.001
All-cause mortality									
As a continuous variable									
PNI	0.847	0.763–0.941	0.002	0.847	0.763–0.941	0.002	0.847	0.763–0.941	0.002
Standardized PNI	0.407	0.231–0.719	0.002	0.407	0.231–0.719	0.002	0.407	0.231–0.719	0.002
As a categorical variable									
T1	Reference			Reference			Reference		
T2	0.410	0.144–1.163	0.094	0.515	0.177–1.492	0.221	0.524	0.179–1.529	0.237
T3	0.160	0.036–0.714	0.016	0.226	0.049–1.047	0.057	0.231	0.050–1.080	0.063
P for trend			0.012			0.119			0.133

For MACE, Model 1 adjusted for age only; Model 2 adjusted for age, smoking, NYHA class III–IV, hypertension, coronary heart disease, stroke, chronic kidney disease, and CHA_2_DS_2_-VASc score; Model 3 adjusted for age, smoking, NYHA class III–IV, hypertension, coronary heart disease, stroke, chronic kidney disease, CHA_2_DS_2_-VASc score, heart rate, total cholesterol, high-density lipoprotein cholesterol, fasting blood glucose, fibrinogen, left ventricular ejection fraction, β-blockers, and anticoagulants. For all-cause mortality, Model 1 did not adjust for covariates; Model 2 adjusted for NYHA class III–IV, hemoglobin, and total cholesterol; Model 3 adjusted for NYHA class III–IV, hemoglobin, total cholesterol, N-terminal pro-brain natriuretic peptide, and β-blockers. MACE, major adverse cardiovascular events; PNI, prognostic nutritional index; CI, confidence interval; HR, hazard ratio.

### 3.5 Predictive value and dose–response relationship

**FIGURE 5:**
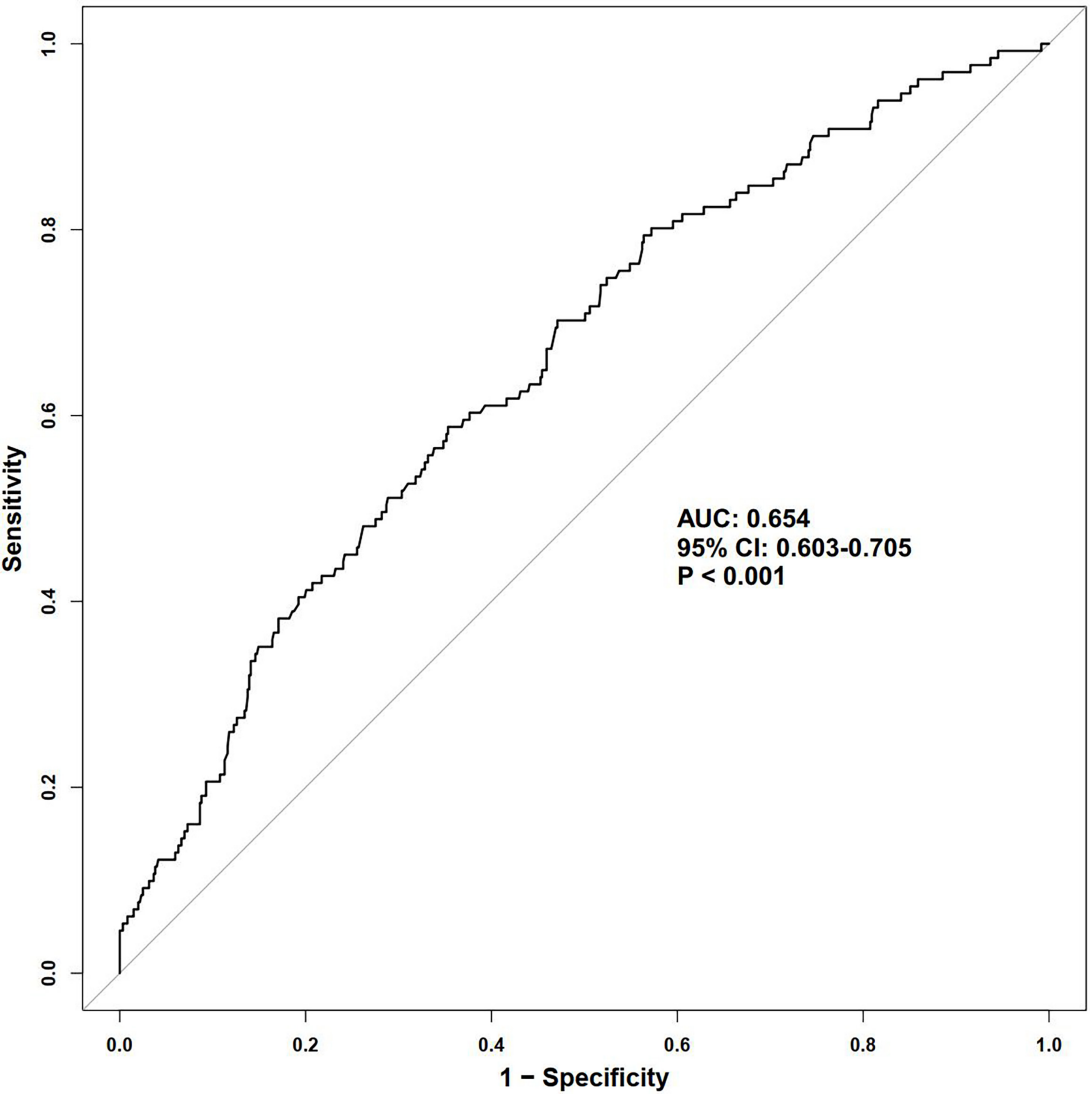
ROC curve analysis evaluating the predictive value of PNI for MACE. ROC, receiver operating characteristic; PNI, prognostic nutritional index; MACE, major adverse cardiovascular events; AUC, area under curve; CI, confidence interval.

**FIGURE 6:**
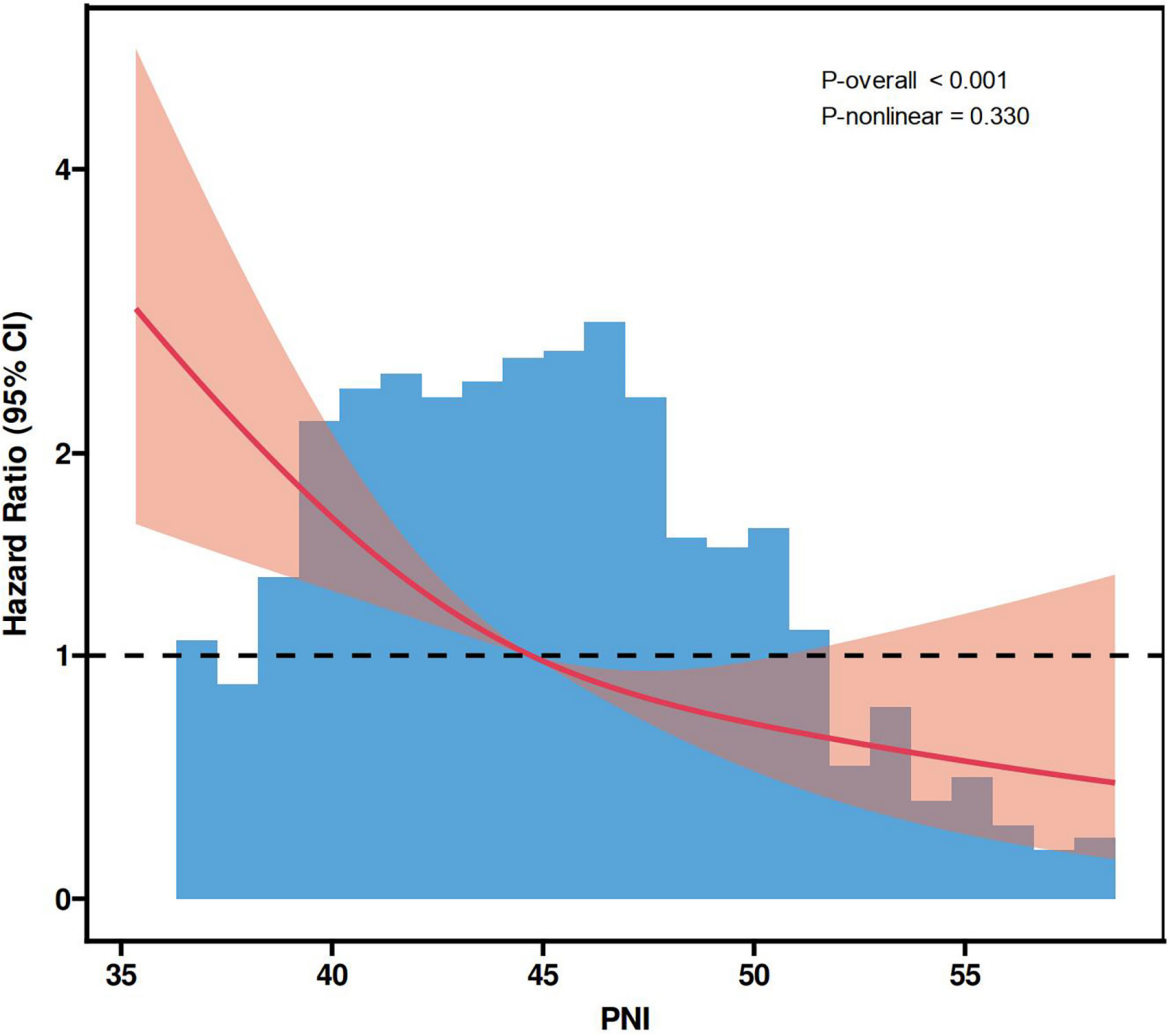
RCS plot evaluating the linear association between PNI and MACE. RCS, restricted cubic spline; PNI, prognostic nutritional index; MACE, major adverse cardiovascular events.

As illustrated in Figure 5, ROC curve analysis demonstrated that PNI showed statistically significant discrimination for MACE, with an AUC of 0.654 (95% CI: 0.603–0.705, *P* < 0.001). As illustrated in Figure 6, RCS plot revealed a significant linear inverse relationship between PNI and MACE (P-overall < 0.001), while no evidence of nonlinearity was detected (P-nonlinear = 0.330).

## 4 Discussion

This study, based on a retrospective cohort of 734 patients with coexisting AF and HFpEF, systematically evaluated the relationship between baseline PNI and the risk of MACE and all-cause mortality. The findings demonstrated that both continuous and categorical forms of PNI were significantly and inversely associated with MACE and all-cause mortality. However, it is important to clarify that statistical significance does not automatically equate to clinical utility. Across different grouping strategies—including median, optimal cutoff, tertiles, and quartiles—higher PNI values consistently corresponded to lower risks of adverse events. Subgroup analyses generally supported the direction of the association across most clinical strata, although certain subgroups did not reach statistical significance and the findings should be interpreted as exploratory. However, it should be noted that in certain subgroups—particularly patients aged ≤ 75 years—the inverse association between PNI and MACE did not reach statistical significance. These findings suggest that the prognostic value of PNI may be relatively attenuated in specific clinical contexts, potentially due to limited event numbers, biological heterogeneity, or residual confounding factors. Such exceptions highlight that the predictive performance of PNI is not uniform across all patient subgroups and warrant cautious interpretation as well as further investigation. ROC and RCS analyses also indicated that PNI had a moderate discriminatory capacity for predicting MACE and showed a linear inverse relationship with event risk. Thus, PNI may serve as an adjunctive marker in comprehensive risk assessment rather than as an independent predictive tool. Taken together, PNI was independently associated with the risk of MACE and all-cause mortality in this cohort, suggesting possible clinical relevance; however, the magnitude of effect and practical implications should be interpreted cautiously given the observational design and moderate discriminatory performance. While the prognostic significance of PNI has been widely reported in various HF and AF populations, the coexistence of AF and HFpEF represents a clinically distinct phenotype characterized by complex bidirectional pathophysiological interactions. AF contributes to atrial remodeling, irregular ventricular response, and hemodynamic instability, whereas HFpEF promotes atrial fibrosis, elevated filling pressures, and systemic inflammation. This intertwined pathophysiology results in a unique risk profile that differs from isolated HF or AF. Therefore, our findings should be interpreted as a targeted validation of PNI within a specific high-risk subgroup rather than a simple reiteration of established associations in broader cardiovascular cohorts.

Recently, increasing attention has been directed toward incorporating nutritional and inflammatory status into HF risk stratification. The PNI, which integrates indicators of nutritional condition and immune function, has shown prognostic relevance across various cardiovascular diseases, including HF. Several previous studies have consistently reported that lower PNI levels are associated with worse clinical outcomes. For example, the AFTER-2 study demonstrated that nutritional indices, including PNI, were significantly associated with adverse cardiovascular outcomes in patients with non-valvular AF (11). Similarly, studies in HF populations have shown that higher PNI levels are associated with reduced risks of mortality and cardiovascular events, including among patients with HFpEF (27–29). Additional investigations in both hospitalized and ambulatory HF cohorts have also demonstrated that low PNI is linked to increased risks of mortality and HF-related rehospitalization, supporting its value as a simple marker for risk stratification in patients with HF or HFpEF (15, 30). Taken together, these studies suggest that PNI may capture a composite risk profile reflecting nutritional status, systemic inflammation, and overall physiological reserve. Rather than representing a disease-specific biomarker, PNI appears to function as an integrative indicator of systemic vulnerability, which may partly explain its consistent association with adverse outcomes across different HF populations. Within this context, our findings further extend the existing literature by specifically evaluating the prognostic significance of PNI in patients with AF and HFpEF, a population characterized by complex comorbidity burden and heterogeneous risk profiles.

Beyond acute HF and HFpEF populations, the prognostic relevance of PNI has also been observed across other HF phenotypes and complex cardiovascular cohorts. Several observational studies have consistently shown that lower PNI levels are associated with higher risks of mortality and HF-related events in patients with HFrEF, device-treated HF populations, and elderly HF cohorts (16, 18, 31). In addition, studies evaluating temporal changes in PNI suggest that improvement in nutritional status during hospitalization may be associated with better clinical outcomes, highlighting the potential importance of dynamic nutritional assessment in HF management (32). Collectively, these findings support the concept that PNI reflects a broader systemic risk profile involving malnutrition, inflammation, and reduced physiological reserve across diverse HF populations. However, the overall evidence base remains heterogeneous. Meta-analytic findings have not been entirely consistent. For example, a systematic review involving HFpEF populations found that while hypoalbuminemia was clearly associated with increased mortality, the pooled association between PNI and mortality did not reach statistical significance, suggesting that population differences, study design, and adjustment strategies may influence observed effects (19). In contrast, a more recent meta-analysis including a broader HF population reported that patients with lower PNI had significantly higher risks of long-term mortality and combined adverse outcomes (33). These discrepancies indicate that the prognostic impact of PNI may be context-dependent and influenced by differences in HF phenotype, baseline nutritional status, comorbidity burden, and analytical approaches. Recent studies have also explored the potential dynamic and clinical implications of PNI. Evidence from device-treated HF populations and arrhythmia cohorts suggests that both baseline PNI levels and longitudinal changes in nutritional status may be associated with HF hospitalization, mortality, and arrhythmia recurrence (20, 34). In addition, pooled analyses from clinical trials indicate that lower PNI is associated with higher risks of composite outcomes across HF phenotypes, while nutritional status may also influence treatment response in certain HFpEF populations (35). Taken together, current evidence suggests that PNI may function as an integrative marker of systemic vulnerability rather than a disease-specific biomarker. Nevertheless, the magnitude and consistency of its prognostic association vary across studies, reflecting heterogeneity in patient populations, disease phenotypes, and methodological approaches. Therefore, our findings should be interpreted as contributing to an evolving body of evidence rather than providing definitive confirmation of a universal prognostic role. In this context, the present study primarily provides subgroup-specific validation of the PNI–outcome association in patients with AF and HFpEF, a population characterized by complex comorbidity burden and intertwined nutritional, inflammatory, and hemodynamic disturbances.

However, evidence regarding PNI in the specific subgroup of patients with AF and HFpEF remains scarce. Against this background, the present study was the first to systematically evaluate the association between PNI and MACE and all-cause mortality in this population, thereby addressing an important gap in the literature. The novelty of our work lies in three aspects: first, focusing on the high-risk subgroup of AF with HFpEF to validate the prognostic role of PNI; second, applying multiple stratification strategies (median, optimal cutoff, tertiles, quartiles) and stepwise multivariable adjustments to ensure the robustness of findings. However, although multiple subgroup and cutoff-based analyses were conducted, these were designed to assess the stability and internal consistency of the association rather than to derive optimal clinical thresholds. Nevertheless, the absence of multiplicity correction may increase the risk of type I error, and these findings should therefore be interpreted cautiously; and third, employing a comprehensive set of statistical approaches—including Kaplan–Meier analysis, Cox regression, ROC curves, and RCS modeling—to assess the predictive performance of PNI and explore dose–response patterns. Taken together, this study extends existing evidence regarding PNI within a specific high-risk phenotype and provides preliminary insight into its potential role in risk stratification, although further validation is required before clinical implementation. From a clinical perspective, PNI may be particularly useful as a simple, low-cost, and readily available nutrition–inflammation indicator for preliminary risk stratification in patients with AF and HFpEF. However, given its moderate discriminatory performance, PNI should not be regarded as a standalone prognostic instrument but rather as a complementary marker within multidimensional risk assessment frameworks. Besides, given that serum albumin and lymphocyte counts are routinely measured in daily practice, PNI can be easily calculated without additional testing burden. This feature may be especially valuable in resource-limited settings or in clinical scenarios where long-term dynamic monitoring of nutritional status is not feasible. In such contexts, PNI could help identify patients who may warrant closer follow-up or more comprehensive risk assessment. However, the practical application of PNI also has important constraints. PNI is influenced by multiple non-cardiac factors, including acute illness, chronic inflammatory conditions, liver function, and overall nutritional intake, and therefore lacks cardiovascular specificity, which may attenuate its prognostic precision in patients with multiple comorbidities. Accordingly, PNI should be interpreted as an adjunctive risk marker rather than a standalone prognostic tool. Notably, in age-stratified analyses, the association between PNI and MACE appeared attenuated in patients aged ≤ 75 years, although the direction of effect remained consistent with the overall cohort. This attenuation may be related to lower event rates and reduced statistical power in younger patients, and age itself may act as an effect modifier given its close relationship with nutritional status, inflammation, frailty, and organ dysfunction. Therefore, age-dependent heterogeneity cannot be excluded, and the strength of association across different age groups should be interpreted cautiously. Further studies with larger samples are needed to clarify potential age-related effect modification. In addition, although our ROC analysis identified an optimal cutoff value of approximately 43–44 (PNI = 43.36) for MACE, the direct clinical application of this threshold should be approached with caution. This cutoff was derived from a single-center retrospective cohort and may vary across populations with different demographic characteristics, disease severity, and treatment patterns. Besides, Although statistically significant, the magnitude of the AUC indicates modest discriminative ability, suggesting that PNI alone may not be sufficient as a standalone predictive tool but could serve as an adjunctive marker in comprehensive risk assessment. Therefore, further large-scale, multicenter prospective studies are needed to validate and refine this cutoff and to determine whether incorporating PNI into clinical decision-making can meaningfully improve patient outcomes. Importantly, the present study does not eliminate prior uncertainty but instead provides subgroup-specific evidence within a defined AF with HFpEF phenotype, which may partially explain why associations observed in broader HF meta-analyses were attenuated or non-significant.

The negative relationship between PNI and MACE risk may reflect the intricate pathophysiological interplay between nutrition, inflammation, and CVD progression. Importantly, in the present cohort, lower PNI levels were consistently associated with markers of more advanced disease severity, including higher NT-proBNP concentrations, worse renal function, higher fibrinogen levels, and a greater proportion of NYHA class III–IV. This graded distribution across PNI tertiles suggests that reduced PNI identifies a clinically vulnerable phenotype characterized by systemic congestion, neurohormonal activation, and multisystem impairment. Firstly, a low PNI typically indicates malnutrition and hypoalbuminemia. In our cohort, the low PNI group demonstrated significantly worse clinical outcomes, including higher levels of NT-proBNP, which correlates with the progression of heart failure. Insufficient serum albumin not only weakens the body’s antioxidant and anti-inflammatory defenses but also correlates closely with the progression of atherosclerosis and endothelial dysfunction (36, 37). In our cohort, patients in the lowest PNI tertile had significantly higher NT-proBNP levels and more advanced NYHA class, indicating more severe hemodynamic stress and symptomatic burden. Given that hypoalbuminemia may reflect chronic catabolic status and impaired protein synthesis, the coexistence of low albumin with elevated cardiac stress markers in our data supports the concept that nutritional depletion parallels HF progression in this population. Secondly, lymphopenia—a key component of the PNI—suggests compromised immune status and persistent low-grade inflammation (38). Lymphocyte count is an integral part of PNI, and we observed that patients with lower PNI had higher levels of inflammatory markers, such as CRP, reinforcing the connection between PNI, immune imbalance, and chronic inflammation. This state of immune imbalance has been recognized as a contributing factor in the progression of HF and the persistence of AF (39). Although specific cytokines were not measured in this study, we observed higher fibrinogen levels and worse renal function in lower PNI strata, both of which are clinically linked to systemic inflammatory activation and endothelial dysfunction. The convergence of these abnormalities within the low-PNI group reinforces the interpretation that PNI may capture a broader inflammatory-metabolic risk state rather than isolated nutritional insufficiency. Thirdly, in patients with HFpEF, chronic inflammation is linked to myocardial fibrosis, diastolic dysfunction, and atrial remodeling—pathological changes that form the substrate for MACE occurrence (40, 41). Within our cohort, the stepwise increase in adverse event rates across decreasing PNI categories, together with the parallel worsening of cardiac functional class and biomarker burden, suggests that PNI is closely aligned with the clinical expression of structural and functional deterioration, even if direct imaging or molecular fibrosis markers were not assessed. Together, these mechanisms suggest that PNI is not merely a nutritional marker but also a potential indicator of immune activity, fibrotic burden, and metabolic stress. The internal consistency observed in our data—where lower PNI coincided with greater cardiac stress, renal impairment, coagulation activation, and symptomatic severity—provides cohort-based clinical support for this integrative interpretation. This multifaceted role may explain its strong prognostic value in patients with HFpEF and coexisting AF. These biologically plausible links lend further support to the use of PNI as a scientifically grounded tool for risk prediction in this population. Nevertheless, the present study was observational and not designed to directly test mechanistic hypotheses. Therefore, the proposed biological pathways linking nutritional status, immune dysregulation, inflammation, myocardial fibrosis, and atrial remodeling remain speculative and require confirmation in translational and mechanistic studies.

Despite the relatively large sample size and comprehensive analyses, this study still has several limitations. First, it was designed as a single-center retrospective cohort, which may introduce selection and information bias, thereby limiting the generalizability of the findings; future multicenter prospective studies are needed for validation. In addition to general selection bias inherent to retrospective designs, this study may also be susceptible to referral bias and institutional practice-pattern effects, as patient characteristics, management strategies, and follow-up protocols may reflect center-specific clinical pathways. Such factors may influence outcome profiles and restrict the extrapolation of findings to other healthcare systems or regions with different practice environments. Moreover, as the sample was drawn from a single institution, the study population may not fully represent the broader AF with HFpEF patient population, potentially limiting external applicability. Second, given that PNI was calculated based on a single-time measurement, the dynamic nature of nutritional and inflammatory status could not be reflected, which may partially limit the robustness of our conclusions. Additionally, no time-dependent covariate analysis was performed due to the lack of longitudinal data on nutritional status and treatment changes in this retrospective cohort. Therefore, all analyses were based on baseline variables, and potential changes during follow-up may not have been captured, which represents a significant methodological limitation. Third, although multiple potential confounders were adjusted for in the statistical models, residual confounding cannot be fully excluded, particularly factors such as frailty, cachexia, sarcopenia, socioeconomic status, dietary patterns, physical activity, chronic inflammatory diseases, liver dysfunction, and other conditions that were not comprehensively captured in this retrospective cohort. These unmeasured variables may influence both PNI levels and clinical outcomes, and therefore may contribute to residual confounding despite multivariable adjustment. Furthermore, unmeasured center-level factors, including variations in therapeutic intensity or resource availability, cannot be completely accounted for and may contribute to residual bias. Importantly, substantial baseline differences were observed across PNI tertiles, including age, NYHA functional class, BMI, renal function, NT-proBNP levels, and inflammatory markers. Notably, NT-proBNP levels were markedly higher in patients with lower PNI, suggesting more advanced HF severity. Although NT-proBNP and other severity indicators were adjusted for in multivariable models, these adjustments may only partially account for the complex relationship between nutritional status and HF severity. Although these variables were incorporated into multivariable-adjusted models, such structural imbalances may indicate residual confounding that cannot be entirely eliminated in an observational study. In addition, differences in medication use across PNI tertiles—including anticoagulants, antihypertensive agents, and lipid-lowering therapies—may reflect potential treatment bias. Patients in lower PNI categories generally exhibited greater disease severity and comorbidity burden, which may have influenced treatment allocation. Although major treatment variables (e.g., β-blockers and anticoagulants) were included in multivariable-adjusted models to mitigate this bias, residual confounding related to treatment intensity, adherence, or duration cannot be completely excluded. Therefore, treatment-related confounding may partially influence the observed associations and should be interpreted cautiously. Fourth, although the ROC analysis showed statistical significance, the AUC value of 0.65 indicates only moderate discriminative ability, suggesting that the predictive performance of PNI for MACE is limited and the robustness of the ROC results should be interpreted cautiously; the relatively low statistical power may also restrict the strength of the conclusions. Moreover, we did not formally evaluate the incremental prognostic value of PNI over established HF risk models or core biomarkers (e.g., NT-proBNP-based risk scores) using standardized approaches such as net reclassification improvement (NRI), integrated discrimination improvement (IDI), or comparative C-statistics. Although additional exploratory single-variable ROC comparisons suggested that PNI demonstrated higher discrimination than individual variables significantly associated with MACE in univariable analyses, such comparisons do not substitute for formal incremental evaluation against validated multivariable risk models. Therefore, whether PNI provides additional prognostic information beyond established HF risk frameworks remains undetermined and requires further investigation. Importantly, we did not perform cost-effectiveness analysis, decision curve analysis (DCA), or any formal clinical utility assessment framework to quantify the net clinical benefit of incorporating PNI into risk stratification. As such, although statistical associations were observed, the practical clinical impact and decision-level usefulness of PNI remain unverified. Future studies should incorporate health-economic evaluation and decision-analytic methodologies to determine whether adding PNI meaningfully improves clinical decision-making and patient outcomes. In addition, the number of mortality events was relatively limited in relation to the covariates included in the fully adjusted model, which may raise concerns about potential overfitting. To mitigate this risk, we adopted a stepwise modeling strategy. Model 1 and Model 2 were constructed in accordance with conventional events-per-variable recommendations, while Model 3 provided more extensive adjustment and should be interpreted primarily as a sensitivity analysis. Although the association between PNI and mortality remained directionally consistent across models, some degree of model instability cannot be entirely excluded. Fifth, while MACE as a composite endpoint reflects overall cardiovascular risk, the underlying mechanisms differ across individual events (e.g., death, stroke, or HF rehospitalization), and the predictive role of PNI for each specific outcome may vary. Moreover, because death may preclude the occurrence of non-fatal events such as stroke or rehospitalization, a competing risk structure may exist within the composite endpoint. In the present study, we applied conventional Cox proportional hazards models and did not perform formal competing risk analyses (e.g., Fine–Gray subdistribution models) or cause-specific hazard modeling for individual components. This was primarily because the main objective was to evaluate the association between PNI and the overall composite event burden rather than to dissect endpoint-specific mechanisms, and because the number of individual non-fatal events was relatively limited, which could compromise model stability in more complex competing risk frameworks. Therefore, the independent contributions of PNI to each component event should be interpreted with caution, and future studies with larger sample sizes should apply competing risk or cause-specific modeling to further clarify differential associations across event types. Sixth, nutritional status, systemic inflammation, HF severity, renal dysfunction, and frailty represent tightly interrelated and overlapping pathophysiological domains in patients with AF and HFpEF. Although multivariable Cox regression models were constructed with adjustment for key clinical and laboratory parameters, conventional statistical adjustment may not fully disentangle these intertwined biological processes. Structural collinearity and shared mechanistic pathways may persist despite adjustment, potentially limiting the ability to isolate the independent contribution of PNI. In particular, low PNI may partly reflect more advanced HF severity or overall systemic deterioration rather than independently driving adverse outcomes, raising the possibility of reverse causation in observational analyses. Therefore, the observed associations should be interpreted as reflecting an integrated risk phenotype rather than definitive evidence of a single independent mechanistic pathway, and causal inference should be approached with caution. To partially address this concern, we additionally performed a sensitivity analysis excluding patients who died within the first 6 months of follow-up; the associations between PNI and outcomes remained directionally consistent, suggesting that the main findings were not solely driven by early severe disease status. Although formal collinearity diagnostics did not demonstrate significant statistical multicollinearity, biological overlap among nutrition, inflammation, and organ dysfunction domains may persist beyond what statistical metrics can fully capture. Seventh, the ROC-derived optimal cutoff for PNI was generated from the same study cohort without external validation. As a data-driven approach, this method may introduce overfitting and optimism bias. Although the main conclusions were supported by continuous-variable analyses, the cutoff-based findings should be interpreted as hypothesis-generating rather than clinically prescriptive. Independent validation in external cohorts is necessary before considering application of any specific threshold in clinical practice. Eighth, no external validation cohort, internal cross-validation, or bootstrap resampling procedures were performed to evaluate the stability of the discriminatory performance or cutoff-based findings. This was primarily because the present study was designed as a single-center observational analysis aimed at examining risk associations rather than developing or validating a formal prediction model. In addition, the absence of an independent dataset within the same study period limited the feasibility of conducting external validation. Consequently, the ROC-related results and derived thresholds may be subject to optimism bias and should be interpreted as exploratory. Future studies incorporating independent cohorts and resampling-based validation techniques are warranted to confirm the robustness and generalizability of these findings. Finally, patients with missing baseline data or those with severe malnutrition were excluded from the analysis, which may have led to underestimation of the true association between PNI and outcomes, and may reduce the applicability of findings to severely malnourished populations. Specifically, exclusion of patients with severe malnutrition may have narrowed the distribution of PNI values by reducing extreme low-range observations, thereby potentially underestimating variability within the cohort. While this approach was intended to minimize confounding from advanced non-cardiovascular conditions, it may also limit external validity, particularly in elderly HFpEF populations where malnutrition is clinically prevalent and prognostically relevant. Therefore, our findings should be interpreted as applicable primarily to AF with HFpEF patients without overt severe malnutrition, and further studies are warranted to evaluate the prognostic role of PNI across a broader spectrum of nutritional status. Taken together, these limitations suggest that the findings should be interpreted with caution. In addition, the lack of external validation and the absence of formal incremental prognostic analyses limit the strength of conclusions regarding clinical applicability. The subgroup analyses were extensive and may have been underpowered in certain strata with limited sample sizes or event counts, resulting in wide confidence intervals and reduced precision of some HR estimates. These analyses were conducted primarily to assess the consistency of associations rather than to provide definitive subgroup-specific conclusions. Although interaction terms were formally tested and corresponding *P*-values were reported, no correction for multiple comparisons was applied, and given the limited statistical power in some strata, both subgroup and interaction findings should be interpreted as exploratory and hypothesis-generating, with caution to avoid overinterpretation.

## 5 Conclusion

In summary, this study demonstrates that PNI is associated with a lower risk of MACE and all-cause mortality in patients with AF and HFpEF. The contribution of this work lies in validating and refining risk stratification within a clinically distinct high-risk phenotype rather than establishing conceptual novelty based solely on disease coexistence. Given its low cost and accessibility, PNI may represent a simple adjunctive marker for identifying patients at higher risk, but its effectiveness in guiding clinical decision-making has not yet been formally established. However, its role should be considered adjunctive, and further multicenter prospective studies are warranted to validate its incremental prognostic value and clinical utility before routine implementation in decision-making algorithms. Further multicenter prospective studies are warranted to validate the incremental prognostic value and to establish the clinical utility of PNI in routine practice.

## Data Availability

The raw data supporting the conclusions of this article will be made available by the authors, without undue reservation.
